# The Impact of Frailty and Geriatric Syndromes on the Quality of Life of Older Adults Receiving Home-Based Healthcare: A Cross-Sectional Survey

**DOI:** 10.3390/healthcare11010082

**Published:** 2022-12-27

**Authors:** Lamprini Tasioudi, Antonia Aravantinou-Karlatou, Savvato Karavasileiadou, Wafa Hamad Almegewly, Emmanouil Androulakis, Christos Kleisiaris

**Affiliations:** 1 Department of Social Sciences, Hellenic Open University, 6335 Patras, Greece; 2 Department of Community Health Nursing, College of Nursing, Princess Nourah bint Abdulrahman University, Riyadh 11671, Saudi Arabia; 3 Mathematical Modeling and Applications Laboratory, Hellenic Naval Academy, 18539 Pireas, Greece; 4 Department of Nursing, Faculty of Health Sciences, Hellenic Mediterranean University, 71410 Heraklion, Greece

**Keywords:** frailty, quality of life, depression, elderly, comorbidity, disability, homecare

## Abstract

Purpose: To identify the effect of frailty and geriatric syndromes on the quality of life (QoL), of older adults receiving home care, taking into consideration their socioeconomic and homebound status, including multi-comorbidities. Patients and Methods: This cross-sectional study enrolled elders aged (≥65) years old, registered members of “Help at Home” programs in the Reference Region of Crete, from March to May 2019. Participants were screened using the WHOQOL-BREF for Quality of Life, geriatric syndromes such as frailty using the SHARE-Frailty Index (SHARE-Fi), the Montreal Cognitive Assessment (MoCA), for cognitive function and the Geriatric Depression Scale (GDS), for the assessment of depression. Results: The mean age of the 301 participants was 78.45 (±7.87) years old. The prevalence of frailty was 38.5%, severe depression 13.6%, cognitive dysfunction 87.8% and severe comorbidity 70.6%. Intriguingly, none of the participants (0%) was identified as free of comorbidity (CCI = 0–1). The overall QoL (ranging from 4–20) of the study participants was 13.24 (±4.09). The bivariate analysis showed that overall QoL significantly differed among older adults with frailty (15.91 vs. 11.56, *p* < 0.001), cognitive dysfunction (15.42 vs. 12.90, *p* < 0.001), depression (14.90 vs. 9.31, *p* < 0.001), and disability in Activities of Daily Living (13.67 vs. 10.67, *p* = 0.002), compared to non-frail, normal cognition and depression, and independent elders, respectively. Multiple linear regression models revealed that frail and depressive elders reported significantly lower QoL (β = −2.65, *p* < 0.001 and (β = −5.71, *p* < 0.001), compared to non-frail and older adults with no depressive symptoms, respectively, despite the fact that this association was not significant for older adults with dementia (β = −2.25, *p* = 0.159), even after adjusting for potential confounding effects (age, gender, comorbidity, homebound status, etc.). Conclusion: frailty and geriatric syndromes including comorbidities are important risk factors for “poor” QoL among older adults receiving home-based healthcare.

## 1. Introduction

Although there is no consensus definition for frailty, it is well-known that it is a complex age-related clinical condition characterized by a decline in physiological capacity across several organ systems, resulting in extreme vulnerability to poor resolution of homeostasis following stress, and is associated with adverse health outcomes [1]. Recent longitudinal studies have shown that frailty is associated with mobility difficulties, hospitalization, death and disability in activities of daily living in terms of physical and cognitive aspects [2,3]. The prevalence of frailty was found to be more common in southern Europe (21.0% in Spain, 14.3% in Italy, 11.3% in Greece, and 9.3% in France), whereas its prevalence was lower than 9.0% in all other countries. However, the prevalence among European countries varies by setting and definition of frailty [4,5].

Geriatric syndromes such as dementia, depression, disability and cognitive impairments are also more prevalent among community-dwelling people aged 65 years old and over, ranging from 8–16% for dementia, 5–40% for depression, 40–50% for cognitive impairments, 9–34% for disability in activities of daily living and 12–58% for visual and hearing impairments [6,7]. In addition, geriatric syndromes affect the quality of life in different ways and levels for each older adult and affect the certain domain of the quality of life in patients receiving homecare [8]. Along the same lines, older adults are not always able to progress in the movement towards well-being and health, with frailty being a decisive limiting factor [9]. 

As both frailty and geriatric syndromes highly coexist in older adults [6], the assessment of the quality of life (QoL) of the elderly is becoming increasingly important in reducing the negative impact of frailty and geriatric syndromes on the quality of life by encouraging older adults to continue their activities of daily living [10]. Most recently, data showed that being healthy, independent, having meaningful relationships and being socially active, as well as being wealth independent were important to all participants in the context of quality of life and sense of well-being [11]. 

The negative impact of frailty on QoL among community-dwelling older adults is well-documented by several studies, showing medium-to-large differences between the groups (frail, pre-frail and robust) [12], and significant effects of frailty on all domains of the QoL [13]. However, only a few home-based studies have documented the influence of frailty and geriatric syndromes on the quality of life of homecare patients [8,14,15], despite the fact that these syndromes are associated with disabilities and disadvantaged social status [16]. 

With regard to homecare patients, the accelerating growth of patients who need homecare creates a need for comprehensive health services and a well-organized health system for allocating home healthcare services [17]. Interestingly, the ‘Help at Home’ program supported by the Greek Ministry of Health and funded by the European Union provides social care, person-centred nursing, and medical care targeting the improvement in QoL by supporting and preventing chronic diseases, social isolation, and institutionalization in older adults who need help at home [18]. 

Therefore, in this survey, the investigation of the existing gap regarding the lack of home-based studies was mainly designed to draw attention to the problem of frailty and geriatric syndromes and their impact on the quality of life of older adults. 

### Aim

Therefore, this study aimed to identify the occurrence of frailty and geriatric syndromes and their impact on the quality of life of older adults receiving home-based healthcare.

## 2. Materials and Methods

### 2.1. Study Design and Population

A cross-sectional study was conducted at 3 randomly selected community-based homecare settings in Crete island, Greece. Initially, we recruited 546 elders 65 years old and over, beneficiaries registered members of “Help at Home” to participate in door-to-door screening for frailty, geriatric syndromes, and quality of life. Data were collected at “Help at Home” programs in the Reference Region of Crete, Greece from March to May 2019. ‘Help at Home’ programs pay attention to older adults with a disadvantaged social status, psychosocial and physical impairments, and/or ‘poor’ family support. 

A total of 245 out of 546 individuals, who did not fully meet the inclusion criteria, were excluded as follows: (1) older adults ≤64 years old; (2) those diagnosed with visual or hearing impairments; (3) those who refused the questionnaire survey; (4) those who were already diagnosed with severe dementia and post-stroke implications; (5) inability to communicate and complete the questionnaire; and (6) an incomplete or unqualified questionnaire. Finally, our statistical analysis involved 301 individuals (response rate of 55.1%). 

### 2.2. Research Instruments

#### 2.2.1. Quality of Life and General Health Assessment

The Greek version of the WHOQOL-BREF was applied to identify the quality of life of our participants. The WHOQOL-BREF is a short form of the original WHOQOL-100 questionnaire developed by the World Health Organization. The validated Greek version comprises 26 questions and the domain scores denote an individual’s perception of quality of life in the following domains: Physical, Psychological, Social Relationships, and Environment. Four (4) additional national items referring to diet, job satisfaction, home life and social life were included for the general health. The validity of the 30-item WHOQOL-BREF Greek version is based on its construct validity (between items and domains and between domain scores) and adequate discriminant validity (between healthy individuals and patient groups) with healthy scoring significantly higher in all four domains, except environment. A 5-point Linkert scale (rate 1–5) obtained total scores for each domain ranging between 4 and 20. Higher scores indicate better quality of life for each domain of each elder. Total scores for each domain range from 4 to 20 [19].

#### 2.2.2. Frailty and Geriatric Syndromes Assessment

Frailty was assessed using the SHARE-Frailty Instrument (SHARE-Fi), a validated screening tool for frailty indicators such as: fatigue; loss of appetite; grip strength; functional difficulties; and physical activity after adjusting for mortality (D factor predicted score R^2^: women 0.97 and men 0.93) [20]. The frailty status (frail; pre-frail and non-frail), was automatically generated after entering the data in an algorithm formula SHARE-FI calculators (one for females and one for males).

Cognitive function was assessed using the Montreal Cognitive Assessment (MoCA), a test used by healthcare providers to evaluate people with memory loss or other symptoms of cognitive decline (sensitivity 0.9), with a Greek-validated version (sensitivity 0.82, specificity 0.90, in a sample of 710 Greek patients), [21]. The MoCA contains 30 questions and checks different types of cognitive or thinking abilities as follows: visuospatial and executive functioning; animal naming; attention; language; abstraction; delayed recall (short-term memory); orientation and education level. Scores ranging from zero to 30. A score of 26 and higher is considered normal.

Geriatric depression was assessed using the Greek version of the Geriatric Depression Scale—short form (GDS-SF), [sensitivity of 92%, specificity of 95%], comprised of 15 items in which the participant is asked to respond by answering “yes” or “no” in reference to how they felt on the day of administration over the past week. A total score ranging between 0–15. A score of 6–10 is suggestive of mild depression and ≥11 is suggestive of severe depression [22]. 

Existed comorbidities were identified using the Charlson Comorbidity Index (CCI). In this study, the online calculator was used to assess adult patients for 15 comorbid chronic conditions, including cerebrovascular disease, diabetes, depression, and cancer [23]. The CCI is a prognostic tool based on the principle that age and the presence and severity of comorbidities increase the likelihood of mortality among patients who receive treatment for chronic illnesses. Based on their CCI scores, patients were grouped into three groups: absence of comorbidity (0–1); moderate risk (2–4); and high risk (≥5), following the suggestions of Huang and his colleagues [24].

#### 2.2.3. Socioeconomic Assessments

The Barthel Scale was used to identify the independence level of elders with a special focus on the disability in Activities of Daily Living (ADL) and performance (reliability Cronbach-a 0.87 and 0.95 universal). The Barthel scale measures the extent to which somebody can function independently and has mobility in their activities of daily living (ADL), i.e., feeding, bathing, grooming, dressing, bowel control, bladder control, toileting, chair transfer, ambulation, and stair climbing. Items are weighted according to the level of nursing care required and are rated in terms of whether individuals can perform activities independently, with some assistance, or are dependent (scored as 10, 5 or 0) [25]. The overall score is ranged between 0 to 20. In the current study, those who self-rated zero to 10 were defined as “dependent” [26].

Homebound status was defined according to the patient’s required assistance when leaving the home, and that when they do it requires a considerable, taxing effort and assessment for potential confounding effects. We classified the homecare older adults into three categories (homebound, semi-homebound and non-homebound), following the suggestions of Ornstein and her colleagues [27].

Socioeconomic status was also estimated by recording the individual characteristics of older adults (gender, age, education, and annual individual income). Participants who had an annual individual income EUR < 4500 (Euros) were considered to live under the “poverty threshold” according to the Hellenic Statistical Authority [28]. 

### 2.3. Ethical Consideration

This study was ethically approved by the Scientific Committee of the MSc Program "Management of Aging and Chronic Diseases" of the Hellenic Open University and the local municipality authorities (“Help at Home” program of Crete (Pr No 297, 2019) in collaboration with the Nursing Department of the Hellenic Mediterranean University and the EIP on AHA’s (A3) Action Group, a community of partners committed to working on specific issues related to active and healthy ageing [29]. Before its implementation, the participants gave their informed consent after having been fully informed that their participation was voluntary, the procedure was anonymous and at any time could withdraw from the study in full compliance with the General Data Protection Regulation (GDPR,) [EU 2016/679] on sensitive personal data. 

### 2.4. Data Analysis

Descriptive statistics were generated as appropriate for each variable. Categorical variables were summarized as frequencies and percentages (n, %), while continuous variables were presented as mean (SD) and median (IQR). Shapiro–Wilk’s test, along with the visual overview of the corresponding histograms, normal Q–Q plots, and box-plots were used to assess the normality of quantitative variables. Baseline differences between quantitative variables and the examined groups were assessed with a t-test for two groups or an Analysis of Variance for three or more groups. Associations between categorical variables were explored with chi-square tests. In addition, to investigate the impact of frailty and geriatric syndromes on quality of life, linear regression models were performed, adjusting for potential confounders (age, gender, education, depression, and comorbidity). Data analysis is presented using unstandardized coefficients (β) and their relevant standard errors (SE), along with 95% confidence intervals (CI). A *p*-value < 0.05 was preset as statistically significant. Data were coded and analyzed using the Statistical Package for Social Science IBM SPSS 24.0.

## 3. Results

### 3.1. Basic Descriptive Characteristics, Geriatric Syndromes and Domains Scores of Quality of Life

Table 1 shows the demographic characteristics of the study participants. The mean age of the 301 older adults was 78.45 ± 7.87 years and 63.1% were female. Moreover, 54.6% of elders had EUR > 4500 annual individual income and 81.4% were uneducated. The quality of life of older adults was reported as follows: physical health was 13.24 (±2.78), mental health was 14.13 (±3.19), social relationships 12.40 (±3.12), environment 14.15 (±2.82) and the overall QoL 13.24 (±4.09). The prevalence of frailty and geriatric syndromes was also presented.

### 3.2. The Quality of Life

Table 2 shows the results of the bivariate analysis presenting the epidemiological differences in the means values of the four domains and the overall QoL, between the (two or more) groups. The data showed that the overall QoL was significantly different among frailty classifications (non-frail, pre-frail, and frail). Similarly, the overall QoL was also ‘poorer’ among patients with cognitive dysfunction (dementia), depression, and comorbidities compared to those with normal cognition, depression and no comorbidities, respectively. As expected, overall QoL was also ‘poorer’ in elders with disability in ADL performance (*p* = 0.002), compared to independent elders. In addition, overall QoL was ‘poorer’ in homebound elders (*p* < 0.001), compared to non-homebound. These associations were observed even though overall QoL was not significantly associated with socioeconomic status (annual individual income, *p* = 0.377 and educational level, *p* = 0.678).

### 3.3. Assessment of the QoL Domains with Respect to Frailty and Geriatric Syndromes

Table 3 shows the effect of frailty, geriatric syndromes, and demographic characteristics on overall QoL. Although univariate analysis shows strong associations between independent variables (frailty, geriatric syndromes, disability, comorbidities, etc.) and the overall QoL (dependent variable), in multivariate analysis (models 2 and 3) these associations are not significant except for frailty and depression. Specifically, frail older adults present a lower quality of life (−2.65, *p* < 0.001) compared to non-frail older adults, suggesting that frailty is a risk factor for a ‘poor’ quality of life. Similarly, older adults with mild depression (−1.65, *p* = 0.001) and severe (−4.40, *p* < 0.001) present the poorest quality of life compared to those with normal depression, respectively. No significant associations were observed as regards QoL and the homebound status of elders (−0.94, *p* = 0.160), socioeconomic status (annual individual income −0.22, *p* = 0.597 and educational level −0.61, *p* = 0.434) after adjusting for homebound status, cardiovascular disease (CVD), gender, age, smoking, annual individual income and educational level. 

Additional analysis was also performed using the mean values of each domain’s quality of life scores and not the mean value of the overall QoL. Along the same lines, assessments of the WHOQOL-BREF were controlled for potential confounding effects and presented in the Supplementary Material (Table S1; Physical Health domain), (Table S2; Psychological Health domain), (Table S3; Social Relationships domain), and (Table S4; Environment domain). 

Specifically, in the physical health domain, frail older adults present lower quality of life (−2.31 grades, *p* < 0.001) compared to non-frail elders, suggesting that frailty and quality of life are significantly associated. Similarly, older adults with severe or mild depression present the poorest quality of life (−1.0, *p* = 0.001, and −2.47, *p* < 0.001) compared to elders with normal depression, respectively. 

Ιn psychological health domain, frail older adults present lower quality of life (−1.74, *p* = 0.001) compared to non-frail older adults, suggesting that frailty and quality of life are significantly associated. Similarly, older adults with severe or mild depression present the poorest quality of life (−1.47, *p* < 0.001, −3.39, *p* < 0.001) compared to older adults with normal depression, respectively.

Furthermore, in the social relationships domain, frail older adults have reported lower quality of life (−1.86, *p* = 0.001) compared to non-frail older adults, suggesting that frailty and quality of life are significantly associated. Similarly, older adults with severe or mild depression present the poorest quality of life (−2.02, *p* < 0.001, −2.46, *p* < 0.001), compared to older adults with normal depression and elders with cognitive dysfunction, (−1.25, *p* = 0.017) when compared to elders with normal cognition, respectively. 

Finally, in the Environment domain, frail older adults have also reported lower quality of life (−2.28, *p* < 0.001) and thus the worst quality of life compared to non-frail older adults, suggesting that frailty and quality of life are significantly associated. Additionally, older adults with severe or mild depression present the poorest quality of life (−2.17, *p* < 0.001, 1.36, *p* < 0.001), compared to older adults with normal depression and elders with cognitive dysfunction also present the poorest QoL (−0.92, *p* = 0.042), compared elders with normal cognition, respectively.

## 4. Discussion

The present study explored the occurrence of frailty and geriatric syndromes and their impact on the Quality of Life of older adults receiving home-based healthcare. In particular, 38.5% of the aged participants were identified as frail, 45.5% as pre-frail and 16% as non-frail. Data analysis showed significant differences among frailty status in variances analysis. Specifically, frail older adults reported poorer overall QoL in comparison to non-frail. We also found that geriatric syndromes, such as dementia and depression, were significantly associated with a ‘poorer’ quality of life among older adults. These associations were also significant when logistic regression models were applied. In general, ‘poor’ overall QoL has been observed in older adults with frailty, cognitive dysfunction (dementia-related impairment), depression, and disability in ADL and homebound older adults even after adjusting for confounding effects. Moreover, socioeconomic status was not significantly associated with ‘poor’ QoL, suggesting that socioeconomic status is not a risk factor for ‘poor’ QoL. 

In agreement with our findings, data from a recent study in Greece that investigated factors influencing the QoL of older adults (aged 60+ years old) demonstrated that demographic characteristics (such as marital status, and children) are among the variables that affect the quality of life of older adults [16]. In addition, findings of a recent study that enrolled registered members of an Open Care Center for older adults and screened older adults with the use of the Tilburg Frailty Indicator (TFI) and WHOQOL-BREF, showed a 54.1% prevalence of frailty and a significant negative relationship between physical frailty, psychological frailty, and all domains of QoL, although these older adults lived independently and were active in a community [13].

Moreover, findings from a longitudinal study conducted on community-dwelling older adults using the TFI and the WHOQOL-BREF showed that physical, psychological and social aspects of frailty are important determinants for improvement in the QoL, emphasizing the importance of a multidimensional approach to frailty assessment [30]. However, data from a cross-sectional study in Spain (the VERISAÚDE study) [31], on community-dwelling older adults, demonstrated that these associations were significant only for the ‘physical’ and ‘Psychological’ domains of the WHOQOL-BREF. Yet, frailty was assessed with the use of Fried’s criteria. Moreover, the quality of life (all domains of the WHOQOL-BREF) was significantly conditioned by the presence of frailty syndrome, both in home environments and in nursing homes.

However, the progression of frailty is associated with the presence of socioeconomic factors, comorbidities and depression [32]. Regardless of the epidemiological approaches and the differences in frailty definitions and settings, it is revealed that frailty is strongly associated with poor QoL. This assumption is established from several studies, despite the fact that different screening tools exist for both frailty and QoL. Particularly, similar findings were observed reporting that frailty was significantly associated with lower grades on the physical and mental health of quality of life, with the use of the SF-36 scale [33]. Likewise, significant associations were also reported with the use of the EuroQol Health Questionnaire, showing that frailty had a negative effect on overall QoL and all domains [34].

As regards geriatric syndromes (depression and cognitive dysfunction), we found that both frailty and depression were significant risk factors for ‘poor’ in all QoL domains. However, cognitive dysfunction was significantly associated only with the ‘Social relationship’ and the ‘Environment’ domains of the WHOQOL-BREF. The associations between depression and dementia-related symptoms have been previously demonstrated by several studies using the WHOQOL-BREF showing lower scores in all domains, especially in patients with severe depression [35]. Furthermore, a study in South Bohemia involved older adults aged 60 years with a special focus on the presence of essential geriatric syndromes (frailty, cognition, sarcopenia and nutritional state), and their influence on the quality of life, which showed that geriatric syndromes mainly affected QoL in terms of the domains of physical health and the psychological state according to the WHOQOL-BREF [8]. On the other hand, a recent study conducted in Taiwan focused on how older adults with chronic diseases can improve their QoL by self-managing, concluding that if elders are aware of their health status they can reduce the risk of frailty and cognitive decline, and subsequently their QoL [36].

Most importantly, comorbidities and disability in ADL performance were not significantly associated with QoL. On the contrary, studies showed that higher comorbidity is significantly associated with lower scores on QoL [37] and health status [38]. This discordance in the associations between the studies can be attributed to the different epidemiologic parameters of the reference populations, as well as the variant assessment tools used in each study.

## 5. Future Implications

To improve the quality of life of older adults and the quality of the services provided, additional steps must be taken in the direction of other European countries’ integrated care schemes. On the other hand, home-based healthcare services are often provided by their family members rather than healthcare professionals (typical caregivers). It is well-known that health professionals working in these programs are not sufficiently trained and qualified in the assessment of frailty and geriatric syndromes, due to a lack of education programs designed to train health professionals in the frailty continuum, provide knowledge about efficacy and effectiveness, and give evidence-based recommendations on curricula development, structure and design [39,40]. It is vital, therefore, to improve the education and training of health professionals in designing person-centered care. It is important to note that providers of vocational educational training need to focus on the development of geriatric protocols that include skills and competencies related to the prevention of both frailty and geriatric symptoms, thereby enhancing the quality of life.

## 6. Limitations

The most important limitation of this study is the low response rate (55%) due to specific characteristics of the sample that participated in the screening (homebound, disabilities, comorbidities, functional limitations, visual or hearing impairments, etc.), thus, possibly not allowing the generalising of our findings compared to other national based-population cohort surveys. On the other hand, no evidence of bias from low response rates has been found when examining relationships between variables in a multivariate analysis. Hence, the results of the univariate analysis (Table 3—model 1) should be exempted from the interpretation of the data based on a common response-rate bias according to Rindfuss and his colleagues [41]. Another limitation could also be the reliability of data, as these have been collected from the participants’ witnesses’ self-reports, which may increase the degree of bias and lead to unsafe conclusions. In addition, given that the WHOQOL-AGE version might be a more applicable tool for this study, this instrument has not been validated for the Greek elderly population yet. However, the 30-item WHOQOL-BREF Greek version is a valid and reliable tool for measuring QoL in healthy and non-healthy populations. Moreover, the cross-sectional nature of the study limits generalization. Within this framework, this study revealed that there are important barriers that should be addressed before assessing QoL in older adults. Nevertheless, it forms baseline data for future local studies. However, it is recommended that future national health surveys measure older adults’ QoL by conducting comprehensive geriatric assessments and involving several processes of care.

## 7. Conclusions

Based on the findings, both frailty and geriatric syndromes are considered to be essential factors that negatively affect the elderly homecare recipients’ quality of life. Therefore, preventive strategies targeting home-based comprehensive geriatric assessment and long-term care management by a geriatric interdisciplinary team (trained and qualified nurses and social workers) who collaborate with the primary care clinician and well-designed approaches (screening, structured visit notes, interventions, etc.), with a special focus on the geriatric syndromes, are considered crucially important. Thus, person-centered nursing interventions tailored to the self-management of geriatric syndromes are necessary for the reduction or delay of disability or risk of frailty in older adults. 

## Figures and Tables

**Table 1:** Descriptive characteristics of the participants (n = 301).

	Mean ± SD, Median (IQR)	
**Age (years)**	78.45 ± 7.87, 79.00 (11.00)	
**QoL**	(range: 4–20)	
Physical health	13.24 ± 2.78, 13.33 (4.00)	
Mental health	14.13 ± 3.19, 14.66 (4.67)	
Social relationships	12.40 ± 3.12, 12.80 (4.00)	
Environment	14.15 ± 2.82, 14.00 (3.50)	
**Overall QoL**	13.24 ± 4.09, 14.00 (6.00)	
	**N**	**%**
**Age**		
≤65–79	153	53.5
≥80	133	46.5
**Gender**		
Male	111	36.9
Female	190	63.1
**Annual individual Income**		
EUR ≤ 4500	133	45.4
EUR > 4500	160	54.6
**Educational Level**		
Uneducated *	245	81.4
Highschool	36	12.0
Bachelor/MSc/PhD	20	6.6
**Frailty status**		
Frail	110	38.5
Pre-frail	130	45.5
Non-frail	46	16
**Cognitive function** ^a^		
Dysfunction (MoCA < 26)	253	87.8
Normal (MoCA 26≥)	35	12.2
**Depression**		
Severe (GDS 11+)	41	13.6
Mild (GDS 6–10)	106	35.2
Normal (GDS 0–5)	154	51.2
**Comorbidity** ^b^		
Severe (CCI ≥ 5)	207	70.6
Mild (CCI 2–4)	86	29.4
Normal (CCI 0–1)	0	0
**Disability in ADL** ^c^		
Dependent (Barthel ≤ 10)	21	7.2
Semi-dependent (Barthel 11–14)	25	8.5
Independent (Barthel 15+)	247	84.3
**Homebound Status** ^d^		
Homebound	54	18.1
Semi-Homebound	48	16.1
Non-Homebound	196	65.8

Notes: Prevalence was given as actual numbers of older adults (N) and percentages (%). * Uneducated refers to having or showing a poor level of formal education (primary school). ^a^ Cognitive Function: MoCA < 26 is indicative of cognitive dysfunction; ^b^ Comorbidity refers to the mean values of the CCI index and not to the actual number of illnesses; ^c^ Disability in ADL refers to the level of functional independence in the domains of personal care and mobility on performing Activities of Daily Living (ADL). Barthel ≤ 10 indicates that the person is dependent or “disabled”); ^d^ Homebound Status refers to the ability of a person to leave home during the last month due to their illnesses. Homebound (able to leave home at least once a week in the last month); Semi-homebound: (able to get home about twice a week with help), Non-homebound: (about twice a week but without help). Abbreviations: GDS, Geriatric Depression Scale; CCI, Charlson Comorbidity index; Barthel Scale-Activities of Daily Living.

**Table 2:** Quality of Life differences (domain’s scores) with respect to frailty, geriatric syndromes, and socio-demographic variables (n = 301).

Quality of Life (WHOQOL-BREF)															
	Physical Health			Mental Health			Social Relationships			Environment			Overall QoL		
	Mean	Sd	*p*-Value	Mean	Sd	*p*-Value	Mean	Sd	*p*-Value	Mean	Sd	*p*-Value	Mean	Sd	*p*-Value
**Frailty**															
Non-frail	15.74	2.66	<0.001	15.91	2.77	<0.001	14.53	2.77	<0.001	16.85	2.49	<0.001	15.91	3.15	<0.001
Pre frail	13.81	2.08		15.07	2.53		13.17	2.91		14.41	2.50		14.10	3.37	
Frail	11.82	2.52		12.60	3.22		10.86	2.78		13.04	2.37		11.56	4.16	
**Cognitive function** ^a^															
Dysfunction	13.04	2.70	<0.001	13.78	3.12	<0.001	12.16	3.05	<0.001	13.86	2.69	<0.001	12.90	3.94	<0.001
Normal (MoCA ≥ 26)	15.21	2.36		16.26	2.49		14.67	2.56		16.12	2.57		15.42	3.71	
**Depression (GDS)**															
Normal	14.42	2.43	<0.001	15.63	2.68	<0.001	13.82	2.76	<0.001	15.31	2.46	<0.001	14.90	3.60	<0.001
Mild	12.54	2.50		13.21	2.86		11.16	2.82		13.27	2.35		12.33	3.78	
Severe	10.62	2.11		10.89	2.31		10.24	2.50		12.06	3.06		9.31	3.11	
**Comorbidity** ^b^															
Mild (CCI ≥ 3)	13.86	2.83	0.030	14.79	3.09	0.058	12.75	3.16	0.297	14.38	2.89	0.547	14.41	3.63	0.004
Severe (CCI ≥ 5)	13.09	2.69		14.02	3.13		12.33	3.13		14.16	2.75		12.91	4.13	
**Disability in ADL** (Barthel) ^c^															
Independent	13.70	2.62	<0.001	14.48	3.15	0.007	12.76	3.14	<0.001	14.55	2.74	<0.001	13.67	3.92	0.002
Semi-dependent	11.75	2.74		13.46	2.84		11.10	2.92		12.70	2.00		12.48	3.70	
Dependent (Barthel < 10)	10.70	2.19		12.44	2.57		10.43	2.24		12.26	2.79		10.67	4.13	
**Homebound status** ^d^															
Non-homebound	14.20	2.47	<0.001	14.91	2.76	<0.001	13.11	3.19	<0.001	14.78	2.73	<0.001	14.09	3.60	<0.001
Semi-homebound	12.06	2.32		13.56	3.23		11.48	2.21		13.83	2.29		12.62	4.54	
Homebound	10.67	2.02		11.55	3.06		10.35	2.27		11.99	2.34		10.44	3.71	
**CVD** *															
Yes	12.94	2.72	0.109	13.75	3.28	0.080	12.15	3.15	0.248	13.82	2.87	0.080	12.55	4.17	0.012
No	13.46	2.78		14.41	3.09		15.57	3.10		14.39	2.73		13.74	3.96	
**Age** (years)															
≥80	12.64	2.74	<0.001	13.70	3.28	0.028	11.90	3.00	0.009	14.02	2.68	0.474	13.07	4.19	0.514
<80	13.77	2.68		14.51	3.05		12.84	3.17		14.26	2.90		13.38	4.00	
**Gender**															
Men	13.79	2.68	0.008	14.52	3.19	0.107	13.12	3.09	0.002	14.81	2.88	0.002	14.00	4.01	0.014
Women	12.92	2.76		13.90	3.16		11.97	3.06		13.76	2.68		12.80	4.08	
**Annual individual Income**															
>4500	13.47	2.82	0.131	14.41	3.19	0.104	12.61	3.19	0.282	14.81	2.95	<0.001	13.46	3.94	0.377
<4500	12.98	2.70		13.80	3.15		12.21	3.10		13.40	2.46		13.03	4.25	
**Smoking**															
Never	13.03	2.82	0.022	13.94	3.27	0.063	12.25	3.09	0.081	13.92	2.69	0.001	13.04	4.11	0.041
Former	13.87	2.56		14.73	3.00		12.92	2.96		14.96	2.70		14.00	3.76	
Current	12.54	2.70		13.35	2.97		11.55	3.62		12.82	3.14		11.92	4.65	
**Educational Level**															
Uneducated	12.97	2.70	0.001	13.96	3.18	0.148	12.17	3.03	0.028	13.97	2.87	0.076	13.14	4.26	0.678
Highschool	14.46	2.87		14.74	3.29		13.15	3.20		14.85	2.23		13.67	3.39	
Bachelor/ MSc/PhD	14.44	2.54		15.10	2.82		13.76	3.68		15.02	2.58		13.70	2.99	

Notes: In this table, the assumption of homogeneity of variance was assessed with the use of Levene’s test. ^a^ Cognitive Function: MoCA < 26 is indicative of cognitive dysfunction; ^b^ Comorbidity refers to the mean values of the CCI index and not to the actual number of illnesses; ^c^ Disability in ADL refers to performing Activities of Daily Living (ADL). Barthel ≤ 10 indicates that the person is dependent or “disabled”. ^d^ Homebound (able to leave home at least once a week in the last month); Semi-homebound: (able to get home about twice a week with help), Non-homebound: (about twice a week but without help). * CVD: Cardiovascular diseases.

**Table 3:** The effect of frailty, geriatric syndromes, and demographic characteristics on Overall QoL.

Linear Regression Models									
	Univariable Models			Multivariable Model 1			Multivariable Model 2		
	β (SE)	95% CI	*p*-Value	β (SE)	95% CI	*p*-Value	β (SE)	95% CI	*p*-Value
**Frailty**									
Pre-frail vs. Non-frail	−1.80 (0.62)	−3.04, −0.56	0.004	−1.18 (0.59)	−2.36, −0.02	0.047	−0.99 (0.61)	−2.21, 0.22	0.109
Frail vs. Non-frail	−4.34 (0.64)	−5.61, −3.08	<0.001	−2.76 (0.67)	−4.07, −1.45	<0.001	−2.65 (0.70)	−4.03, −1.27	<0.001
**Cognitive function** ^a^ (Moca)									
Dysfunction vs. Normal (<26 vs. ≥26)	−2.52 (0.71)	−3.92, −1.14	<0.001	−0.55 (0.64)	−1.82, 0.71	0.389	−0.94 (0.67)	−2.25, 0.37	0.159
**Depression** (GDS)									
Mild vs. Normal	−2.56 (0.45)	−3.46, −1.67	<0.001	−1.58 (0.46)	−2.49, −0.67	0.001	−1.65 (0.47)	−2.58, −0.73	0.001
Severe vs. Normal	−5.59 (0.63)	−6.84, −4.34	<0.001	−4.17 (0.63)	−5.42, −2.91	<0.001	−4.40 (0.66)	−5.71, −3.09	<0.001
**Comorbidity** ^b^ (CCI)									
Severe (CCI ≥ 5) vs. Mild	−1.50 (0.51)	−2.51, −0.49	0.004	−0.78 (0.43)	−1.63, 0.06	0.069	−0.83 (0.50)	−1.82, 0.15	0.098
**Disability in ADL** ^c^ (Barthel)									
Semi-dependent vs. Independent	−1.19 (0.83)	−2.84, 0.45	0.153	0.81 (0.74)	−0.65, 2.27	0.278	0.76 (0.75)	−0.73, 2.26	0.319
Dependent vs. Independent	−3.01 (0.90)	−4.78, −1.23	0.001	−0.11 (0.87)	−1.82, 1.61	0.905	−0.26 (0.88)	−2.00, 1.47	0.765
**Homebound status** ^d^									
Semi-homebound vs. Non-homebound	−1.46 (0.62)	−2.68, −0.24	0.019	0.45 (0.62)	−0.77, 1.67	0.471	0.37 (0.64)	−0.88, 1.64	0.560
Homebound vs. Non-homebound	−3.64 (0.59)	−4.81, −2.48	<0.001	−1.18 (0.63)	−2.41, 0.05	0.061	−0.94 (0.67)	−2.25, 0.37	0.160
**CVD** *									
Yes vs. No	−1.19 (0.47)	−2.12, −0.26	0.012	−	−	−	−0.30 (0.47)	−1.23, 0.63	0.523
**Age** (years)									
≥80 vs. <80	−0.31 (0.47)	−1.24, 0.62	0.514	−	−	−	0.29 (0.44)	−0.58, 1.17	0.510
**Gender**									
Men vs. Women	1.20 (0.48)	0.25, 2.15	0.014	−	−	−	0.33 (0.49)	−0.63, 1.31	0.500
**Annual individual Income**									
>4500 vs. <4500	0.43 (0.48)	−0.52, 1.36	0.377	-	-	-	−0.22 (0.41)	−1.03, 0.59	0.597
**Smoking**									
Current vs. Never	−1.12 (0.83)	−2.77, 0.52	0.181	-	-	-	−0.46 (0.79)	−2.03, 1.09	0.555
Former vs. Never	0.95 (0.51)	−0.07, 1.97	0.068	-	-	-	−0.09 (0.50	−1.08, 0.89	0.847
**Educational Level**									
Highschool vs. Uneducated	0.52 (0.73)	−0.92, 1.96	0.475	-	-	-	−0.82 (0.61)	−2.03, 0.38	0.182
Bachelor/MSc/PhD vs. Uneducated	0.56 (0.95)	−1.32, 2.43	0.560	-	-	-	−0.61 (0.78)	−2.14, 0.92	0.434

Notes: ^a^ Cognitive Function: MoCA < 26 is indicative of cognitive dysfunction; ^b^ Comorbidity refers to the mean values of the CCI index and not to the actual number of illnesses; ^c^ Disability in ADL refers to performing Activities of Daily Living (ADL). Barthel ≤ 10 indicates that the person is dependent or “disabled”; ^d^ Homebound status refers to the ability of a person to leave home during the last month due to its illnesses. Homebound (able to leave home at least once a week in the last month); Semi-homebound: (able to get home about twice a week with help), Non-homebound: (about twice a week but without help). Abbreviations: β’ unstandardized coefficients (SE): standard error; CI: Confidence Intervals. Overall QoL is controlled as a dependent variable in this linear model meaning. Example: In the relation “Frail vs. non-frail” it is expected reduction in Overall QoL score (-4.34 grades), this also means that lower scores as worse Overall QoL. * CVD: Cardiovascular diseases.

## Data Availability

The data presented in this study are available on request from the corresponding author. The data are not publicly available due to privacy restrictions.

## Supplementary Materials

The following supporting information can be downloaded at: https://www.mdpi.com/article/10.3390/healthcare11010082/s1, Table S1: The effect of frailty, geriatric syndromes, and other social demographic factors on Physical Health (QoL), Table S2: The effect of frailty, geriatric syndromes, and other social demographic factors on Psychological Health (QoL), Table S3: The effect of frailty, geriatric syndromes, and other social demographic factors on Social Relationships (QoL) and Table S4: The effect of frailty, geriatric syndromes, and other social demographic factors on Environment (QoL).
