# Supplementary material for: The Impact of Frailty and Geriatric Syndromes on the Quality of Life of Older Adults Receiving Home-Based Healthcare: A Cross-Sectional Survey
Source: Healthcare (Basel). 2022 Dec 27;11(1):82. doi: 10.3390/healthcare11010082 (PMC9819361; doi:10.3390/healthcare11010082)
Supplement: Supplementary file 1 [file healthcare-11-00082-s001.zip › healthcare-2068169-supplementary.pdf]

healthcare

MDPI

Table S1. The effect of frailty, geriatric syndromes, and other social demographic factors on Physical Health (QoL).

	Linear regression models								
	Univariable models			Multivariable model 1			Multivariable model 2		
	β (SE)	95% CI	p-value	β (SE)	95% CI	p-value	β (SE)	95% CI	p-value
**Frailty**									
Pre frail vs Non-frail	−1.93 (0.40)	−2.73, −1.13	<0.001	−1.37 (0.36)	−2.09, −0.64	<0.001	−1.34 (0.38)	−2.10, −0.58	0.001
Frail vs Non-frail	−3.92 (0.41)	−4.74, −3.11	<0.001	−2.35 (0.41)	−3.16, −1.54	<0.001	−2.31 (0.43)	−3.17, −1.45	<0.001
**Cognitive function**[a] (Moca)									
Dysfunction vs Normal (<26 vs ≥26)	−2.16 (0.48)	−3.11, −1.21	<0.001	−0.45 (0.39)	−1.23, 0.32	0.248	−0.44 (0.41)	−1.26, 0.37	0.289
**Depression** (GDS)									
Mild vs Normal	−1.87 (0.30)	−2.47, −1.27	<0.001	−0.95 (0.28)	−1.50, −0.38	0.001	−1.00 (0.29)	−1.58, −0.42	0.001
Severe vs Normal	−3.80 (0.42)	−4.64, −2.97	<0.001	−2.46 (0.39)	−3.23, −1.69	<0.001	−2.47 (0.41)	−3.29, −1.66	<0.001
**Comorbidity**[b] (CCI)									
Severe (CCI≥5) vs Mild	−0.76 (0.35)	−1.45, −0.075	0.030	−1.71 (0.26)	−0.69, 0.35	0.519	−0.08 (0.31)	−0.69, 0.53	0.793
**Disability in ADL**[c] (Barthel)									
Semi-dependent vs Independent	−1.95 (0.54)	−3.02, −0.87	<0.001	0.07 (0.45)	−0.82, 0.98	0.868	−0.02 (0.47)	−0.96, 0.91	0.968
Dependent vs Independent	−2.99 (0.59)	−4.15, −1.82	<0.001	−0.40 (0.53)	−1.46, 0.65	0.452	−0.49 (0.55)	−1.58, 0.59	0.370
**Homebound status**[d]									
Semi-homebound vs Non-homebound	−2.14 (0.38)	−2.89, −1.38	<0.001	−0.84 (0.38)	−1.59, −0.08	0.029	−0.63 (0.40)	−1.42, 0.15	0.115
Homebound vs Non-homebound	−3.53 (0.36)	−4.25, −2.81	<0.001	−1.86 (0.38)	−2.61, −1.09	<0.001	−1.58 (0.41)	−2.40, −0.76	<0.001
**CVD***									
Yes vs No	−0.51 (0.32)	−1.15, 0.12	0.109	-	-	-	−0.14 (0.29)	−0.72, 0.43	0.624
**Age** (years)									
≥80 vs <80	−1.12 (0.31)	−1.74, −0.51	<0.001	-	-	-	−0.39 (0.27)	−0.94, 0.15	0.154
**Gender**									
Male vs Female	0.87 (0.32)	0.23, 1.51	0.008	-	-	-	−0.33 (0.30)	−0.64, 0.57	0.915
**Annual individual Income**									
>4500 vs <4500	0.49 (0.32)	−0.14, 1.14	0.131	-	-	-	−0.28 (0.25)	−0.78, 0.23	0.278
**Smoking**									
Current vs Never	−0.48 (0.56)	−1.60, 0.62	0.388	-	-	-	−0.46 (0.49)	−1.43, 0.52	0.355
Former vs Never	0.84 (0.34)	0.15, 1.53	0.017	-	-	-	0.11 (0.31)	−0.50, 0.72	0.718
**Educational Level**									
Highschool vs Uneducated	1.49 (0.48)	0.54, 2.45	0.002	-	-	-	0.26 (0.38)	−0.49, 1.02	0.492
Bachelor/MSc/PhD vs Uneducated	1.47 (0.63)	0.23, 2.71	0.020	-	-	-	0.46 (0.48)	−0.49, 1.42	0.340

Notes: [a]Cognitive Function: MoCA<26 is indicative of cognitive dysfunction; [b]Comorbidity refers to the mean values of the CCI index and not to the actual number of illnesses; [c]Disability in ADL refers to performing Activities of Daily Living (ADL). Barthel ≤10 indicates that the person is dependent or "disabled"); [d]Homebound status refers to the ability of a person to leave home during the last month due to their illnesses. Homebound (able to leave home at least once a week in the last month); Semi-homebound: (able to get home about 2 times a week with help), Non-homebound: (about 2 times a week but without help). Abbreviations: β' unstandardized coefficients(SE): standard error; CI: Confidence Intervals. Physical Health (QoL) is controlled as a dependent variable in this linear model meaning. Example: In the relation "Frail vs. non-frail" it is expected reduction of Overall QoL score (−4.34 grades), this also means that lower scores as worse Overall QoL. *CVD: Cardiovascular diseases.

Table S2. The effect of frailty, geriatric syndromes, and other social demographic factors on Psychological Health (QoL).

Linear regression models									
	**Univariable models**			**Multivariable model 1**			**Multivariable model 2**		
	**β (SE)**	**95% CI**	**p-value**	**β (SE)**	**95% CI**	**p-value**	**β (SE)**	**95% CI**	**p-value**
**Frailty**									
Pre frail vs Non-frail	−0.84 (0.49)	−1.80, 0.12	0.087	−0.18 (0.44)	−1.07, 0.70	0.685	−0.23 (0.34)	−1.17, 0.70	0.627
Frail vs Non-frail	−3.31 (0.50)	−4.30, −2.32	<0.001	−1.76 (0.50)	−2.75, −0.77	0.001	−1.74 (0.54)	−2.81, −0.68	0.001
**Cognitive function**[a] (Moca)									
Dysfunction vs Normal (<26 vs ≥26)	−2.47 (0.55)	−3.56, −1.39	<0.001	−1.02 (0.48)	−1.97, −0.06	0.037	−0.88 (0.51)	−1.89, 0.12	0.087
**Depression** (GDS)									
Mild vs Normal	−2.41 (0.34)	−3.09, −1.74	<0.001	−1.48 (0.34)	−2.17, −0.80	<0.001	−1.47 (0.36)	−2.19, −0.76	<0.001
Severe vs Normal	−4.74 (0.47)	−5.67, −3.80	<0.001	−3.57 (0.48)	−4.51, −2.62	<0.001	−3.39 (0.51)	−4.40, −2.38	<0.001
**Comorbidity**[b] (CCI)									
Severe (CCI≥5) vs Mild	−0.76 (0.40)	−1.55, 0.03	0.058	−0.17 (0.32)	−0.81, 0.46	0.589	−0.05 (0.38)	−0.80, 0.71	0.905
**Disability in ADL**[c] (Barthel)									
Semi-dependent vs Independent	−1.09 (0.65)	−2.29, 0.25	0.117	0.79 (0.56)	−0.31, 1.90	0.156	0.74 (0.58)	−0.41, 1.90	0.206
Dependent vs Independent	−2.04 (0.70)	−3.42, −0.65	0.004	0.51 (0.65)	−0.78, 1.81	0.435	0.50 (0.68)	−0.83, 1.84	0.458
**Homebound status**[d]									
Semi-homebound vs Non-homebound	−1.34 (0.46)	−2.27, −0.42	0.004	−0.12 (0.46)	−1.05, 0.79	0.791	−0.11 (0.49)	−1.08, 0.86	0.831
Homebound vs Non-homebound	−3.35 (0.44)	−4.23, −2.48	<0.001	−1.43 (0.47)	−2.36, −0.50	0.003	−1.46 (0.51)	−2.47, −0.45	0.005
**CVD***									
Yes vs No	−0.65 (0.37)	−1.38, 0.08	0.080	-	-	-	−0.19 (0.36)	−0.90, 0.52	0.596
**Age** (years)									
≥80 vs <80	−0.81 (0.36)	−1.53, −0.09	0.028	-	-	-	−0.22 (0.34)	−0.90, 0.45	0.515
**Gender**									
Male vs Female	0.61 (0.38)	−0.13, 1.36	0.107	-	-	-	−0.13 (0.38)	−0.89, 0.61	0.716
**Annual individual Income**									
>4500 vs <4500	0.61 (0.37)	−0.13, 1.34	0.104	-	-	-	0.06 (0.31)	−0.56, 0.69	0.838
**Smoking**									
Current vs Never	−0.58 (0.65)	−1.87, 0.69	0.370	-	-	-	−0.42 (0.61)	−1.62, 0.78	0.494
Former vs Never	0.78 (0.40)	−0.01, 1.58	0.053	-	-	-	0.21 (0.38)	−0.54, 0.97	0.583
**Educational Level**									
Highschool vs Uneducated	0.77 (0.56)	−0.34, 1.88	0.173	-	-	-	0.26 (0.38)	−0.49, 1.02	0.492
Bachelor/MSc/PhD vs Uneducated	1.13 (0.73)	−0.32, 2.58	0.126	-	-	-	0.46 (0.48)	−0.49, 1.42	0.340

Notes: [a]Cognitive Function: MoCA<26 is indicative of cognitive dysfunction; [b]Comorbidity refers to the mean values of the CCI index and not to the actual number of illnesses; [c]Disability in ADL refers to performing Activities of Daily Living (ADL). Barthel ≤10 indicates that the person is dependent or "disabled"); [d]Homebound status refers to the ability of a person to leave home during the last month due to their illnesses. Homebound (able to leave home at least once a week in the last month); Semi−homebound: (able to get home about 2 times a week with help), Non-homebound: (about 2 times a week but without help). Abbreviations: β'unstandardized coefficients(SE): standard error; CI: Confidence Intervals. Psychological Health (QoL) is controlled as a dependent variable in this linear model meaning. Example: In the relation "Frail vs. non-frail" it is expected reduction of Overall QoL score (−4.34 grades), this also means that lower scores as worse Overall QoL. *CVD: Cardiovascular diseases.

Table S3. The effect of frailty, geriatric syndromes, and other social demographic factors on Social Relationships (QoL).

Linear regression models									
	**Univariable models**			**Multivariable model 1**			**Multivariable model 2**		
	**β (SE)**	**95% CI**	**p-value**	**β (SE)**	**95% CI**	**p-value**	**β (SE)**	**95% CI**	**p-value**
**Frailty**									
Pre frail vs Non-frail	−1.36 (0.48)	−2.32, −0.40	0.005	−0.61 (0.46)	−1.52, 0.31	0.193	−0.42 (0.48)	−1.38, 0.53	0.385
Frail vs Non-frail	−3.67 (0.49)	−4.65, −2.69	<0.001	−1.98 (0.51)	−3.01, −0.96	<0.001	−1.86 (0.55)	−2.93, −0.77	0.001
**Cognitive function**[a] **(**Moca)									
Dysfunction vs Normal (<26 vs ≥26)	−2.51 (0.54)	−3.57, −1.45	<0.001	−1.36 (0.49)	−2.34, −0.38	0.007	−1.25 (0.52)	−2.28, −0.22	0.017
**Depression** (GDS)									
Mild vs Normal	−2.67 (0.34)	−3.35, −1.98	<0.001	−1.96 (0.35)	−2.67, −1.25	<0.001	−2.02 (0.36)	−2.74, −1.29	<0.001
Severe vs Normal	−3.58 (0.48)	−4.53, −2.63	<0.001	−2.57 (0.49)	−3.54, −1.59	<0.001	−2.46 (0.52)	−3.49, −1.43	<0.001
**Comorbidity**[b] (CCI)									
Severe (CCI≥5) vs Mild	−0.42 (0.40)	−1.21, 0.37	0.297	0.32 (0.33)	−0.33, 0.98	0.331	0.60 (0.39)	−0.17, 1.37	0.126
**Disability in ADL**[c] (Barthel)									
Semi-dependent vs Independent	−1.66 (0.64)	−2.92, −0.39	0.010	0.17 (0.57)	−0.96, 1.31	0.761	0.04 (0.59)	−1.13, 1.22	0.940
Dependent vs Independent	−2.33 (0.69)	−3.67, −0.95	0.001	−0.47 (0.67)	−1.81, 0.86	0.487	−0.54 (0.69)	−1.90, 0.82	0.432
**Homebound status**[d]									
Semi-homebound vs Non-homebound	−1.63 (0.46)	−2.56, −0.71	0.001	−0.12 (0.48)	−1.08, 0.83	0.801	0.08 (0.50)	−0.90, 1.08	0.863
Homebound vs Non-homebound	−2.76 (0.44)	−3.64, −1.88	<0.001	−0.96 (0.49)	−1.93, −0.01	0.48	−0.61 (0.52)	−1.64, 0.42	0.242
**CVD***									
Yes vs No	−0.42 (0.36)	−1.14, 0.29	0.248	-	-	-	−0.46 (0.36)	−1.18, 0.27	0.219
**Age** (years)									
≥80 vs <80	−0.94 (0.35)	−1.64, −0.23	0.009	-	-	-	−0.61 (0.35)	−1.30, 0.076	0.081
**Gender**									
Male vs Female	1.14 (0.36)	0.42, 1.87	0.002	-	-	-	0.68 (0.38)	−0.08, 1.44	0.080
**Annual individual Income**									
>4500 vs <4500	0.39 (0.37)	−0.33, 1.13	0.282	-	-	-	−0.32 (0.33)	−0.95, 0.31	0.320
**Smoking**									
Current vs Never	−0.71 (0.64)	−1.96, 0.56	0.275	-	-	-	−0.90 (0.62)	−2.13, 0.32	0.147
Former vs Never	0.67 (0.39)	−0.11, 1.45	0.091	-	-	-	−0.32 (0.39)	−1.09, 0.45	0.412
**Educational Level**									
Highschool vs Uneducated	0.97 (0.55)	−0.11, 2.06	0.079	-	-	-	−0.14 (0.48)	−1.09, 0.80	0.761
Bachelor/MSc/PhD vs Uneducated	1.58 (0.72)	0.16, 2.99	0.029	-	-	-	0.87 (0.61)	−0.32, 2.07	0.153

Notes: [a]Cognitive Function: MoCA<26 is indicative of cognitive dysfunction; [b]Comorbidity refers to the mean values of the CCI index and not to the actual number of illnesses; [c]Disability in ADL refers to performing Activities of Daily Living (ADL). Barthel ≤10 indicates that the person is dependent or "disabled"); [d]Homebound status refers to the ability of a person to leave home during the last month due to their illnesses. Homebound (able to leave home at least once a week in the last month); Semi-homebound: (able to get home about 2 times a week with help), Non-homebound: (about 2 times a week but without help). Abbreviations: β'unstandardized coefficients(SE): standard error; CI: Confidence Intervals. Social Relationships (QoL) are controlled as a dependent variable in this linear model meaning. Example: In the relation "Frail vs. non-frail" it is expected reduction of Overall QoL score (−4.34 grades), this also means that lower scores as worse Overall QoL. *CVD: Cardiovascular diseases.

Table S4. The effect of frailty, geriatric syndromes, and other social demographic factors on Environment (QoL).

Linear regression models									
	**Univariable models**			**Multivariable model 1**			**Multivariable model 2**		
	**β (SE)**	**95% CI**	**p-value**	**β (SE)**	**95% CI**	**p-value**	**β (SE)**	**95% CI**	**p-value**
**Frailty**									
Pre frail vs Non-frail	−2.43 (0.42)	−3.26, −1.60	<0.001	−1.87 (0.41)	−2.67, −1.06	<0.001	−1.82 (0.42)	−2.64, −0.99	<0.001
Frail vs Non-frail	−3.81 (0.43)	−4.65, −2.96	<0.001	−2.58 (0.46)	−3.47, −1.68	<0.001	−2.28 (0.47)	−3.22, −1.35	<0.001
**Cognitive function**[a] **(**Moca)									
Dysfunction vs Normal (<26 vs ≥26)	−2.27 (0.48)	−3.21, −1.31	<0.001	−0.83 (0.44)	−1.70, 0.03	0.060	−0.92 (0.45)	−1.81, −0.04	0.042
**Depression** (GDS)									
Mild vs Normal	−2.04 (0.32)	−2.66, −1.41	<0.001	−1.25 (0.32)	−1.88, −0.63	<0.001	−1.36 (0.32)	−1.99, −0.74	<0.001
Severe vs Normal	−3.25 (0.44)	−4.12, −2.38	<0.001	−2.17 (0.43)	−3.03, −1.31	<0.001	−2.17 (0.45)	−3.05, −1.28	<0.001
**Comorbidity**[b] (CCI)									
Severe (CCI≥5) vs Mild	−0.21 (0.35)	−0.92, 0.49	0.547	0.36 (0.29)	−0.21, 0.94	0.216	0.47 (0.33)	−0.19, 1.14	0.161
**Disability in ADL**[c] (Barthel)									
Semi-dependent vs Independent	−1.85 (0.57)	−2.97, −0.74	0.001	−0.67 (0.51)	−1.67, 0.34	0.192	−0.65 (0.51)	−1.67, 0.35	0.204
Dependent vs Independent	−2.29 (0.61)	−3.49, −1.08	<0.001	−0.73 (0.59)	−1.91, 0.44	0.222	−0.69 (0.59)	−1.86, 0.48	0.248
**Homebound status**[d]									
Semi-homebound vs Non-homebound	−0.95 (0.41)	−1.77, −0.13	0.024	0.67 (0.42)	−0.16, 1.52	0.113	0.77 (0.43)	−0.08, 1.63	0.075
Homebound vs Non-homebound	−2.79 (0.40)	−3.58, −2.01	<0.001	−0.94 (0.43)	−1.78, −0.09	0.030	−0.59 (0.45)	−1.48, 0.29	0.189
**CVD***									
Yes vs No	−0.57 (0.32)	−1.21, 0.07	0.080	-	-	-	−0.36 (0.31)	−0.99, 0.26	0.253
**Age** (years)									
≥80 vs <80	−0.23 (0.32)	−0.87, 0.40	0.474	-	-	-	0.02 (0.30)	−0.58, 0.61	0.953
**Gender**									
Male vs Female	1.05 (0.33)	0.39 (1.69)	0.002	-	-	-	−0.06 (0.33)	−0.71, 0.59	0.860
**Annual individual Income**									
>4500 vs <4500	1.41 (0.32)	0.77, 2.04	<0.001	-	-	-	0.89 (0.27)	0.34, 1.44	0.002
**Smoking**									
Current vs Never	−1.07 (0.56)	−2.19, 0.04	0.058	-	-	-	−0.74 (0.53)	−1.79, 0.32	0.171
Former vs Never	1.03 (0.35)	0.34, 1.72	0.003	-	-	-	0.45 (0.33)	−0.21, 1.12	0.181
**Educational Level**									
Highschool vs Uneducated	0.87 (0.49)	−0.10, 1.85	0.079	-	-	-	0.14 (0.41)	−0.68, 0.96	0.739
Bachelor/MSc/PhD vs Uneducated	1.05 (0.65)	−0.23, 2.32	0.107	-	-	-	0.41 (0.52)	−0.62, 1.45	0.433

Notes: [a]Cognitive Function: MoCA<26 is indicative of cognitive dysfunction; [b]Comorbidity refers to the mean values of the CCI index and not to the actual number of illnesses; [c]Disability in ADL refers to performing Activities of Daily Living (ADL). Barthel ≤10 indicates that the person is dependent or "disabled"); [d]Homebound status refers to the ability of a person to leave home during the last month due to their illnesses. Homebound (able to leave home at least once a week in the last month); Semi-homebound: (able to get home about 2 times a week with help), Non-homebound: (about 2 times a week but without help). Abbreviations: β′ unstandardized coefficients (SE): standard error; CI: Confidence Intervals. Environment (QoL) is controlled as a dependent variable in this linear model meaning. Example: In the relation "Frail vs. non-frail" it is expected reduction of Overall QoL score (−4.34 grades), this also means that lower scores as worse Overall QoL. *CVD: Cardiovascular diseases.
